# Meta-analysis of bone mineral density in adults with phenylketonuria

**DOI:** 10.1186/s13023-024-03223-9

**Published:** 2024-09-12

**Authors:** Júlio C. Rocha, Álvaro Hermida, Cheryl J. Jones, Yunchou Wu, Gillian E. Clague, Sarah Rose, Kaleigh B. Whitehall, Kirsten K. Ahring, André L. S. Pessoa, Cary O. Harding, Fran Rohr, Anita Inwood, Nicola Longo, Ania C. Muntau, Serap Sivri, François Maillot

**Affiliations:** 1 NOVA Medical School/Faculdade de Ciências Médicas (NMS/FCM), Universidade NOVA de Lisboa, Lisboa, Portugal; 2 Reference Centre of Inherited Metabolic Diseases (RC-IMD), Centro Hospitalar Universitário de Lisboa Central, Lisboa, Portugal; 3 CINTESIS@RISE, Nutrition and Metabolism, NOVA Medical School, Faculdade de Ciências Médicas, NMS, FCM, Universidade NOVA de Lisboa, Lisboa, Portugal; 4 University of Santiago de Compostela, Santiago de Compostela, Spain; 5 HCD Economics, Knutsford, UK; 6 BioMarin Pharmaceutical Inc, Novato, CA USA; 7 Clinic for PKU, Copenhagen University Hospital, Copenhagen, Denmark; 8 Federal University of Ceará - UFC, Fortaleza, CE Brazil; 9 Hospital Infantil Albert Sabin, Fortaleza, CE Brazil; 10 Oregon Health & Science University, Portland, OR USA; 11 Met Ed Consultants, Boulder, CO USA; 12 Queensland Lifespan Metabolic Medicine Service, Queensland Children’s Hospital, South Brisbane, QLD Australia; 13 University of Utah School of Medicine, Salt Lake City, UT USA; 14 University Children’s Hospital, University Medical Center Hamburg-Eppendorf, Hamburg, Germany; 15 Hacettepe University, Ankara, Turkey; 16 Internal Medicine Department and Reference Center for Inherited Metabolic Disease, University of Tours, Tours, France

**Keywords:** Phenylketonuria, Phenylalanine, Bone, Bone mineral density, Osteopenia, Osteoporosis, Meta-analysis, Z-score, Diet, Diet adherence

## Abstract

**Background:**

Lifelong management of phenylketonuria (PKU) centers on medical nutrition therapy, including dietary phenylalanine (Phe) restriction in addition to Phe-free or low-Phe medical foods/protein substitutes. Studies have reported low bone mineral density (BMD) in mixed-age PKU populations, possibly related to long-term Phe restriction. Therefore, a meta-analysis investigating BMD specifically in adults with PKU was conducted.

**Methods:**

Studies reporting BMD-related outcomes were identified from a systematic literature review evaluating somatic comorbidities experienced by adults with PKU on a Phe-restricted diet (searched February 1, 2022, updated November 1, 2023). Risk of study bias was assessed (Scottish Intercollegiate Guidelines Network checklists). The primary outcome of the meta-analysis was pooled mean BMD Z-scores of different bones. Secondary outcomes were the prevalence of low BMD Z-scores at pre-specified thresholds. Subgroup analyses of mean BMD Z-scores (decade of study publication, controlled versus uncontrolled blood Phe levels, gender) were conducted.

**Results:**

BMD-related data from 4097 individuals across 10 studies rated as at least acceptable quality were included. Mean BMD Z-scores were statistically significantly lower compared with an age-matched control or reference (non-PKU) population, across bones, but still within the expected range for age (> -2.0): lumbar spine (seven studies, *n* = 304), -0.63 (95% confidence interval (CI): -0.74, -0.52); femoral neck (four studies, *n* = 170), -0.74 (95% CI: -1.25, -0.22); radius (three studies, *n* = 114), -0.77 (95% CI: -1.21, -0.32); total body (four studies, *n* = 157), -0.61 (95% CI: -0.77, -0.45). The small number of observations in the subgroup analyses resulted in a high degree of uncertainty, limiting interpretation. Estimated prevalence of BMD Z-scores ≤ -2.0 was 8% (95% CI: 5%, 13%; four studies, *n* = 221) and < -1.0 was 42% (95% CI: 35%, 51%; five studies, *n* = 144).

**Conclusions:**

Adults with PKU had lower BMD Z-scores than the reference (non-PKU) population but < 1 in 10 were below the expected range for age. The low number of studies prevents identification of which population characteristics are most impacting BMD.

This meta-analysis was supported by BioMarin Pharmaceutical Inc., Novato, CA and is registered with the Research Registry (reviewregistry1476).

**Supplementary Information:**

The online version contains supplementary material available at 10.1186/s13023-024-03223-9.

## Background

Phenylalanine hydroxylase (PAH) deficiency (OMIM# 261600), known commonly as phenylketonuria (PKU), is an autosomal recessive metabolic disorder caused by pathogenic variants in the gene encoding PAH. Impaired PAH function impairs conversion of the essential amino acid phenylalanine (Phe) to tyrosine [1]. Accumulation of Phe in the blood and the brain has toxic effects, blocking transport of other large neutral amino acids into the brain, including tyrosine and tryptophan, via competition at the L-type amino acid transporter 1 [1].

Untreated PKU results in poor neurological outcomes [1, 2]. Therefore, the goal of treatment is to achieve and maintain appropriate blood Phe levels. The American College of Medical Genetics guidelines recommend a blood Phe level of 120–360 µmol/L for all patients [3]; European guidelines recommend 120–360 µmol/L for patients < 12 years of age and 120–600 µmol/L for patients ≥ 12 years of age [4].

Medical nutrition therapy (MNT) [3] for PKU is lifelong and restricts the natural intake of protein, replacing it with a Phe-free, amino acid-based medical food to supplement the reduced protein intake, and provide a source of energy and other nutrients. Supplements might include modified low-protein foods, Phe-free medical food beverages, Phe-free amino acid mixture, medical foods derived from glycomacropeptide, and protein substitutes [3].

It is difficult for all individuals with PKU to achieve and maintain a Phe-restricted diet, particularly into adolescence and adulthood [2, 5]. Maintaining blood Phe levels within recommended ranges, both during and after childhood is important to achieve the best long-term outcomes [2]. Elevated blood Phe levels may be due to low Phe tolerance (the amount of daily Phe intake that an individual can consume without increasing blood Phe levels above the recommended range), which is linked to disease severity, and in turn, linked to mutation status and level of functional PAH. This explains how even with active management, Phe levels remain elevated in some individuals [2, 5, 6].

Individuals with PKU can also present with secondary health problems; healthcare claims-based studies suggest a higher prevalence of somatic comorbidities versus the respective general population [7, 8].

Abnormal bone status has been a concern for a long time in individuals with PKU [4, 9]. Currently, it is unclear whether low bone mineral density (BMD) in those with PKU is a direct consequence of the disease, the result of a Phe-restricted diet [10] or due to reliance on low-Phe amino acid supplementation, which can increase urinary calcium and magnesium excretion [11, 12]. Mineral bone disease, where bone strength deteriorates, increasing the risk of osteopenia, osteoporosis, and ultimately fracture [13], has been reported in individuals with PKU [14].

BMD contributes to bone strength and measuring BMD Z-scores is one method used to help determine an individual’s bone density status, where the individual Z-score is the number of standard deviations by which BMD differs from that expected for age and sex. The International Society for Clinical Densitometry Official Position (ISCD OP) considers a BMD Z-score ≤ -2.0 as below the expected range for age, and a BMD Z-score > -2.0 as within the expected range for age, in females prior to menopause and males younger than 50 years of age [15]. Other BMD Z-score thresholds are also considered to be clinically informative, with a BMD Z-score of < -2.5 considered indicative of a secondary cause of osteoporosis [16–18] and a BMD Z-score of < -1.0 and ≥ -2.5 considered indicative of osteopenia [16, 18, 19]; however, the ISCD OP states that in some populations (e.g. children and adolescents, males under the age of 50 years) osteoporosis cannot be diagnosed using BMD only [15, 20].

A previous meta-analysis reported lower mean BMD Z-scores for various bones in individuals with PKU on a Phe-restricted diet versus the respective general population, but BMD Z-scores were still within the expected range for age, based on the ISCD OP definition [9]. Similarly, a systematic literature review (SLR), without meta-analysis also reported lower mean BMD Z-scores in those with PKU on a Phe-restricted diet versus a reference population, but again, within the expected range for age [21]. Both studies reported BMD in mixed-age PKU populations, and so the inclusion of both children and adults limited the interpretation of BMD status by an individual’s age. This, coupled with the use of different thresholds to evaluate BMD status, means the evidence base has not reached a consensus on BMD status in individuals with PKU.

A meta-analysis has been conducted to investigate BMD outcome measures in adults with PKU, and to explore the impact of the Phe-restricted diet (including impact of adherence to diet, most often assessed by blood Phe level control) on BMD. This meta-analysis included studies identified in a broader SLR that was conducted to evaluate published evidence on the somatic comorbidities experienced by adults with PKU; these results have been reported separately in Whitehall et al. [22]. BMD was assessed in adults with PKU on a Phe-restricted diet versus a reference (non-PKU) population using reported BMD Z-scores for various bones. The prevalence of low BMD Z-scores at pre-specified thresholds was assessed in adults with PKU on a Phe-restricted diet.

## Methods

This meta-analysis is reported according to Meta-analysis Of Observational Studies in Epidemiology reporting guidelines [23].

### Systematic literature review methodology

Data for inclusion in the meta-analysis were identified from the broader SLR; full details of the eligibility criteria for inclusion of studies, information sources, search strategy, and selection process for the SLR have been reported separately. Briefly, eligibility criteria were established using the Population, Intervention, Comparator, Outcomes and Study designs (PICOS) framework [24] and included peer-reviewed observational studies (cohort, case–control, cross-sectional, surveys) and clinical trials in adults ≥ 16 years of age (or classified as sexually mature) with confirmed, or described as having, PKU. Studies carried out exclusively in a population < 16 years of age were excluded. Eligible studies included those evaluating a Phe-restricted diet versus no form of therapeutic intervention (non-PKU controls or reference values) reporting BMD-related data. Single cohort studies in individuals with PKU, who were untreated, narrative review articles, letters, editorials, commentaries, therapy recommendations, clinical guidelines, congress abstracts, and non-peer-reviewed literature, were excluded.

Literature was retrieved via the PubMed® interface using search terms (Additional file 1: Table S1) relevant to the PICOS. No date restrictions were applied to the search, thus publications in English from MEDLINE earliest coverage to November 1, 2023 [25] were retrieved and assessed by researchers with at least one postgraduate qualification. Medical Subject Headings (MeSH) search terms and free-text terms for BMD, bone mineral content, osteoporosis, and bone loss were included as part of the full systematic search string designed to identify data on somatic comorbidities. Records eligible for data extraction in the SLR were identified using a two-stage screening process whereby each record was screened once at each stage: first-pass screening of title and abstract, and second-pass screening of full texts of all records considered potentially eligible after first pass. Reference lists of relevant SLRs and meta-analyses (retrieved as part of the systematic literature search) were reviewed to identify any additional papers of interest (via backwards citation searching).

### *Meta*-analysis methodology

#### Study selection

All studies included in the SLR were assessed for inclusion of BMD-related data relevant to the objectives of the meta-analysis, that is, BMD Z-scores for specific bones (e.g. lumbar spine, femoral neck) in adults with PKU on a Phe-restricted diet versus a reference (non-PKU) population, and the prevalence of low BMD Z-scores (or prevalence of osteopenia/osteoporosis) in adults with PKU on a Phe-restricted diet. Studies were included regardless of whether adherence to diet was assessed or reported. Studies reporting on a mixed treatment population (e.g. in which some individuals were treated with a pharmacologic intervention, rather than or in addition to a Phe-restricted diet) were excluded from the meta-analysis if BMD data were not reported separately by treatment type. To ensure adequacy of the search and selection process in identifying all relevant studies, those identified for inclusion in the meta-analysis of BMD Z-scores were cross-checked against the previously reported Demirdas et al. [9] meta-analysis.

#### Data collection and data outcomes

Data extraction from eligible studies was conducted by one reviewer into a pre-designed data extraction spreadsheet (Microsoft Excel®) and included characteristics of study populations (e.g. age, gender), study design, and interventions, as well as outcome definitions and results (i.e. BMD Z-scores and standard deviations, prevalence data). Extracted data were checked for accuracy (of the extraction) by a second reviewer.

#### Study risk of *bias* assessment

Risk of bias was assessed for all studies included in the meta-analysis using either the Scottish Intercollegiate Guidelines Network (SIGN) methodology checklist 3 for cohort studies (18 questions) or the SIGN methodology checklist 4 for case–control studies (15 questions) [26], depending on the study design. The SIGN checklists were selected to be consistent with the risk of bias assessment conducted in the meta-analysis reported by Demirdas et al. [9]. SIGN checklists are based on the Grading of Recommendations, Assessment, Development, and Evaluation approach [27] and include questions related to the research question, selection, enrollment, follow-up of study participants, assessment of outcomes, confounding, and statistical analysis, to determine whether a study should be considered of high, acceptable, or unacceptable quality. The risk of bias assessment was conducted manually by two reviewers working independently to ensure consensus was reached regarding the level of bias for each study. Outcomes were visualized via traffic light plots using Microsoft Excel®.

#### Effect measures

The effect measure for the primary objective was the BMD Z-score. As defined by the ISCD OP [15], and to align with the BMD meta-analysis reported previously by Demirdas et al. [9], BMD Z-scores > -2.0 were interpreted as being within the expected range for age.

The effect measure for the secondary objective was the proportion of individuals with a low BMD Z-score at a pre-specified threshold. To align with Demirdas et al. [9] and the ISCD OP [15], the prevalence of BMD Z-scores ≤ -2.0 (so below the expected range for age) was analyzed, as well as the prevalence of BMD Z-scores < -1.0, a Z-score indicating possible osteopenia and/or osteoporosis [19]; < -1.0 and ≥ -2.5, a Z-score indicating possible osteopenia [19]; and < -2.5, a Z-score that may indicate a secondary cause of osteoporosis [16–18].

#### Synthesis methods

BMD Z-scores represent a continuous dataset; therefore, outcome measures are presented as a mean value, with standard deviation (SD), or as a median value, with a range (min, max) or interquartile range (range from Q1 to Q3). Mean and SD were used to determine the standardized mean difference (SMD). Prevalence of low BMD Z-scores represents a proportional (binary) dataset thus outcome measures are presented as the number of events out of the total sample or as a percentage of the total sample. These data are already standardized across studies and do not require further transformation.

For those studies where data were presented as a median value, with a range (min, max) or interquartile range (range from Q1 to Q3), the median values were transformed into mean values using published methodology [28]. The mean difference (MD) was defined as the mean BMD Z-score from the population of individuals with PKU on a Phe-restricted diet. The SMD, defined as the MD divided by the pooled SD, was then calculated to standardize the effect size across studies. The generated SMD is equivalent to Cohen’s d, which can overestimate the true effect size, particularly with small sample sizes; in such cases Hedges’ g correction was used to transform Cohen’s d.

Data were synthesized using packages (e.g. meta, metafor, rmeta) in R software. Statistical significance was set to 5% (*p* < 0.05) for all analyses and 95% confidence intervals (CIs) were used to express uncertainty. Both fixed effects and random effects models were used to apply weights to each individual study in the meta-analysis and to estimate the effect size of interest per analysis. The degree of heterogeneity between studies was then used to guide the decision on which effect size to consider, with the fixed effects model adopted in situations of low to moderate heterogeneity between studies [29] and the random effects model adopted in situations of high heterogeneity between studies [29].

#### Assessment of heterogeneity

Heterogeneity was assessed visually using forest plots. The Cochran’s Q test (with a significance level of 0.05) and I^2^ statistic (with the following thresholds: 0–40% might not be important; 30–60% may represent moderate heterogeneity; 50–90% may represent substantial heterogeneity; 75–100% may represent considerable heterogeneity), was applied to assess the significance and the degree of statistical heterogeneity respectively [30].

#### Assessment of publication *bias*

A symmetrical scattering of points on the funnel plot indicates no publication bias and an asymmetric scattering of points indicates potential publication bias.

#### Subgroup analyses

Subgroup analysis of lumbar spine BMD Z-scores was conducted to explore the impact of population and study characteristics on the overall effect estimate. Analysis by decade of publication of studies included in the primary objective analysis was conducted as a proxy for reported improvements in Phe-restricted dietary supplements over time, to evaluate the impact of diet on the overall effect estimate.

A subgroup analysis was performed to investigate the impact of blood Phe-level control (as defined in individual studies and considered a proxy for treatment adherence) on BMD Z-scores.

Subgroup analysis by gender was also conducted, as BMD and bone mineral content tend to be higher in males than in females [31]. Selection of the appropriate model (fixed effects or random effects) for study weighting was based on heterogeneity at the overall level (considering both subgroups) as recommended by Borenstein et al. [32]. Subgroup analysis of BMD Z-scores across other bones was also conducted where data were available.

#### Sensitivity analyses

Studies that included children or adolescents (< 18 years old or of an age defined by the study as adults) as well as adults ≥ 18 years of age, and did not stratify outcomes by participant age, were eligible for inclusion in the SLR. Therefore, the meta-analysis of BMD Z-scores included data from studies in which some individuals were < 18 years of age (and therefore not strictly considered as being of adult age). A sensitivity analysis was conducted to include only studies of individuals ≥ 18 years of age to determine the impact of including those < 18 years of age on the BMD Z-scores effect estimate compared with the overall analysis.

Studies reporting on the prevalence of osteopenia and osteoporosis were eligible for inclusion in the meta-analysis of the prevalence of low BMD Z-scores, where it was assumed that individuals with osteopenia or osteoporosis would have a BMD Z-score of < -1.0 and ≥ -2.5 (osteopenia) [16, 18, 19] or < -2.5 (osteoporosis) [16–18]. A sensitivity analysis was conducted to determine the impact of excluding data from these individuals on the effect estimate compared with the overall analysis.

## Results

### Study selection and study characteristics

**Fig. 1:**
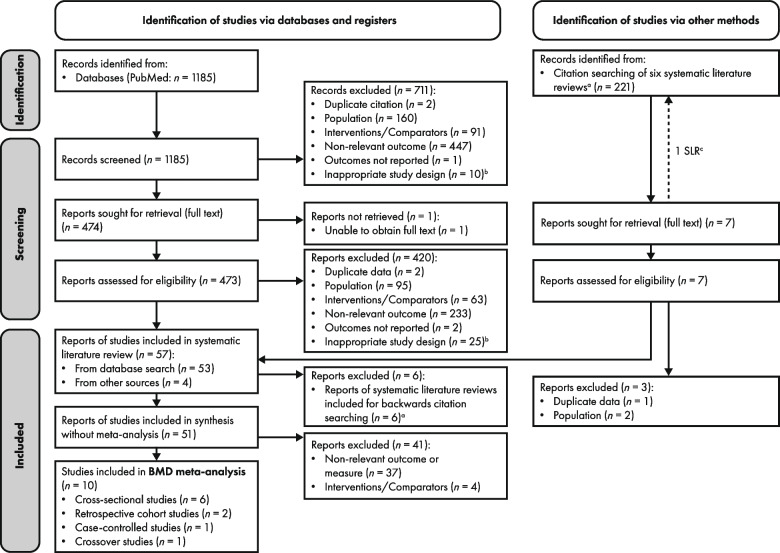
PRISMA diagram of article flow. ^a^ Six systematic reviews were identified via the database search and used for backwards citation searching only; ^b^ Includes studies that did not present outcomes data in a way that answered one or more of the pre-specified research questions; ^c^ One of the studies identified by backwards citation searching of a published SLR was itself an SLR that was subsequently used for further backwards citation searching. BMD, bone mineral density; PRISMA, Preferred Reporting Items for Systematic Reviews and Meta-Analyses

Figure 1 shows the results of the study selection process for the SLR and meta-analysis. Of the 51 studies included in the synthesis without meta-analysis, reported in the separate SLR publication, 10 studies reported BMD-related data (either BMD Z-scores and/or prevalence of low BMD Z-scores/osteopenia/osteoporosis) relevant to the objectives of this meta-analysis, and so were included in this study; the other 41 studies reported non-relevant outcomes/measures (*n* = 37) or interventions (*n* = 4) and were excluded. For example, four studies were excluded [33–36] because a proportion of the PKU population was receiving sapropterin dihydrochloride [33–36] or pegvaliase [35] either instead of, or in addition to, a Phe-restricted diet, and results were not presented separately for the population on dietary therapy alone (study authors were not contacted to request this information).

Characteristics of the 10 studies identified for inclusion in the meta-analysis are presented in Table 1. Seven studies were conducted in Europe, one study in the Middle East, and two in the United States. Most individuals with PKU were early treated, and disease severity ranged from mild hyperphenylalaninemia (HPA) to classical PKU (severe disease). Characteristics of the PKU populations in each study, including mean age and age range, and mean or median baseline Phe concentrations; intervention and comparator; definitions of Phe control/diet-adherence or compliance; and BMD-related data, included in the meta-analysis, are presented in Table 2. Adults (≥ 16 years of age or classified as sexually mature) with PKU and on a Phe-restricted diet were compared with an age-matched control population or a reference (non-PKU) population and, in line with the meta-analysis objectives, all studies reported BMD Z-scores and/or the prevalence of low BMD Z-scores/osteopenia/osteoporosis.

**Table 1:** Summary of characteristics of studies (*n* = 10)

Study; geographic location	Design	PKU population				Aim
		**Sample size, treatment, and subgroup definitions**	**Mean** **age ± SD** **(unless** **stated), years**	**Gender (M/F)**	**Disease severity**	
Adamczyk et al. 2011 [37]; Europe (Poland)	Cross-sectional study	- 45 individuals with early treated PKU on a Phe-restricted diet^a^ ◦ Subgroup 1: individuals with recommended^b^ blood Phe levels (*n* = 15) ◦ Subgroup 2a: individuals (Tanner stage 5^c^) with recommended^b^ blood Phe levels (*n* = 18) ◦ Subgroup 2b: individuals (Tanner stage 5^c^) with blood Phe levels above the recommended^b^ level (*n* = 12)	- All PKU: 13.8 ± 5.2 ◦ Subgroup 1: 7.1 ± 1.4; range, 4.9–10.2 ◦ Subgroup 2a: 17.6 ± 2.2; range, 12.5–21.6 ◦ Subgroup 2b: 16.8 ± 2.1; range, 14.6–21.9	- M: *n* = 25, 56% - F: *n* = 20, 44%	- NR	- Assess bone metabolism and the muscle–bone relationship in children, adolescents, and young adults with PKU
Burton et al. 2018 [8]; North America (USA)	Case-controlled study	- 3691 individuals with PKU (unclear whether participants were on a Phe-restricted diet)	- 34.6 ± 14.3; range, 20–NR	- M: *n* = 1341, 36% - F: *n* = 2350, 64%	- NR	- Determine the prevalence of comorbid conditions in adults with PKU
Choukair et al. 2017 [38]; Europe (Germany)	Cross-sectional study	- 56 individuals (49 adults; 7 adolescents) with early treated PKU: ◦ 33 individuals (29 adults; 4 adolescents) were on a Phe-restricted diet supplemented with amino acids ◦ 16 individuals (14 adults; 2 adolescents) did not follow a diet or take amino acid supplementation ◦ 3 individuals (all adults) received amino acid supplementation only ◦ 4 individuals (3 adults; 1 adolescent) followed a Phe-restricted diet exclusively	- All PKU: 26.0 ± 8.9; range, 11.8–41.5 ◦ Adults: 27.9 ± 7.91; range 14.8–41.5 ◦ Adolescents: 13.1 ± 1.07; range 11.8–14.7	- M: *n* = 16, 39% - F: *n* = 40, 71%	- HPA^d^: 2% - Mild PKU^d^: 27% - Classical PKU^d^: 71%	- Assess bone characteristics and the muscle–bone relationship in adolescents and adults with PKU
de Groot et al. 2012 [39]; Europe (The Netherlands)	Cross-sectional study	- 53 individuals with early treated PKU on a Phe-restricted diet	- 16.7 ± 9.1; range 2–35	- M: *n* = 25, 47% - F: *n* = 28, 53%	Based on pre-treatment Phe concentrations: - Mild HPA: 43% - Mild PKU: 17% - Moderate PKU: 10% - Severe PKU: 30% Based on Phe tolerance at 24 months: - Mild HPA: 0% - Mild PKU: 16% - Moderate PKU: 72% - Severe PKU: 12%	- Investigate the relationship between lumbar BMD and biochemical parameters in individuals with PKU
Lage et al. 2010 [16]; Europe (Spain)	Cross-sectional study	- 47 individuals with PKU on a Phe-restricted diet ^e^ (mix of early and late-diagnosis) ◦ Group 1: 6–10 years: *n* = 8 ◦ Group 2: 11–18 years: *n* = 17 ◦ Group 3: 19–42 years: *n* = 22	- All PKU: range, 6–42 ◦ Group 1: 6–10 years: 8.4 ± 1.3 ◦ Group 2: 11–18 years: 13.7 ± 2.6 ◦ Group 3: 19–42 years: 29.3 ± 6.6	- All PKU: M: *n* = 30, 64%; - F: *n* = 17, 36% ◦ Group 1: 6–10 years: M: *n* = 5, 63%; F: *n* = 3, 38% ◦ Group 2:11–18 years: M: *n* = 11, 65%; F: *n* = 6, 35% ◦ Group 3: 19–42 years: M: *n* = 14, 64%; F: *n* = 8, 36%	Based on Phe concentration^g^: All PKU: - Mild PKU: 21% - Moderate: 23% - Classical: 55%	- Evaluate the relationship between fatty acid profile and bone health in individuals with PKU
Lubout et al. 2020 [10]; Europe (UK, France, The Netherlands, Poland, Spain)	Retrospective cohort study	- 183 individuals with early treated PKU on a Phe-restricted diet	- Median: 32; range, 19–53	- M: *n* = 77, 42% - F: *n* = 106, 58%	Based on Phe concentration^g^: - Mild HPA: 10% - Mild PKU: 22% - Classical PKU: 68%	- Assess the prevalence of low BMD and define possible risk factors in adults with early treated PKU
Modan-Moses et al. 2007 [40]; Middle East (Israel)	Cross-sectional study	- 31 individuals with early treated PKU; individuals were adherent or non-adherent^h^ to a Phe-restricted diet (number of individuals in each group was unclear)	- All: 25 ± 5.3; range, 19–41 - Adherent to diet: 24.0 ± 3.8 - Non-adherent to diet: 25.7 ± 6.2	- M: *n* = 13, 42% - F: *n* = 18, 58%	- Classical PKU^i^: 100%	- Evaluate BMD in young adults with PKU at peak bone mass and relate BMD to nutritional parameters
Pérez-Dueñas et al. 2002 [19]; Europe (Spain)	Retrospective cohort study	- 28 individuals with PKU on a Phe-restricted diet^j^ (15/28 individuals had a late diagnosis) - 11 individuals relaxed diet at age 7–8 years for a mean of 10 years - 1 individual was not on a Phe-restricted diet owing to ulcerous colitis	- 18^ k^; range, 10–33	- M: *n* = 11, 39% - F: *n* = 17, 61%	- NR	- Assess bone mineralization in individuals (≥ 10 years of age) and relate BMD to nutritional parameters and vitamin D treatment
Stroup et al. 2017 [12]; North America (USA)	Crossover study	- 8 individuals with early treated PKU on a Phe-restricted diet plus either their preferred Phe-free amino acid based medical food or glycomacropeptide medical food, during the study period	- 27.3 ^j^; range, 16–35	- M: *n* = 4, 50% - F: *n* = 4, 50%	- Classical PKU: 50% - Variant PKU: 50%	- Evaluate dietary acid load with AA-MF compared with GMP-MF
Zeman et al. 1999 [41]; Europe (Czech Republic)	Cross-sectional study	- 44 individuals with early treated PKU on a Phe-restricted diet^l^	- 16.1^ k^; range, 6–29	- M: *n* = 19, 43% - F: *n* = 25, 57%	- Classical PKU: 100%	- Assess BMD (lumbar and total) in children, adolescents, and young adults with PKU and relate BMD to nutritional parameters

*AA-MF* amino acid medical foods, *BMD* Bone mineral density, *DXA* Bone density scan, *F* Female, *GMP-MF* Glycomacropeptide medical foods, *HPA* Hyperphenylalaninemia, *M* Male, *NR* Not reported, *Phe* Phenylalanine, *PKU* Phenylketonuria, *PUFA* Polyunsaturated fatty acid, *SD* Standard deviation

^a^Phe-free formula combined with small, controlled amounts of natural protein

^b^The recommended blood Phe levels in treated individuals < 12 years old is 2–6 mg/dL and 2–10 mg/dL for individuals ≥ 12 years old, whereas the level for healthy people is < 2.8 mg/dL

^c^Tanner stage 5 indicates sexual maturity

^d^Method of classification not reported

^e^Phe-free amino acid mixture, depending on individual dietary Phe tolerance, which was averaged over a period of 6 months (non-natural protein source contributed 15–93% of daily protein intake), individuals received neither *n*-3 nor *n*-6 PUFA supplementation from the time their treatment was implemented

^f^Classical PKU (Phe > 1200 µmol/L), moderate PKU (Phe 600–1200 µmol/L), mild PKU (Phe 360–600 µmol/L)

^g^PKU severity classification based on mean Phe concentration the year before the most recent DXA assessment: classical PKU (≥ 1200 µmol/L), mild PKU (> 600– < 1200 µmol/L), mild HPA (≤ 600 µmol/L)

^h^ Based on self-report (non-adherent individuals had been off-diet for 7.4 SD: 4.9 years)

^i^Phe concentration on diagnosis was > 20 mg/dL

^j^Phe-free formula

^k^No SD or median reported

^l^Phe-free amino acid mixture

**Table 2:** Summary of patient characteristics and BMD data included in the meta-analysis

Study	Sample size	Mean age ± SD (unless stated) of sample included in the MA, years	Intervention and diet adherence/blood Phe level control	Comparator	Outcome measures reported in the study, by bone location, and used in the MA
Adamczyk et al. 2011 [37]	Total sample of 45 individuals with PKU Number of individuals included in the MA: 30 - Subgroup 2a: - *N* = 18 - Subgroup 2b: - *N* = 12	Subgroup 2a: 17.6 ± 2.2; range, 12.5–21.6 Subgroup 2b: 16.8 ± 2.1; range, 14.6–21.9	Early treatment with a Phe-restricted diet (Phe-free) Blood Phe monitored at least every 2 months, which serves for modification of diet Recommended blood Phe levels: Individuals < 12 years 2–6 mg/dL Individuals ≥ 12 years 2–10 mg/dL Blood Phe higher in Subgroup 2b versus Subgroup 2a: 21.86 SD ± 2.21 mg/dL^a^ versus 11.11 SD ± 1.36 mg/dL^a^	No explicit comparator BMD Z-score implicitly compares with a reference value based on healthy, age, and gender matched controls	- Mean BMD Z-scores: 1. Lumbar spine 2. Total body
Burton et al. 2018 [8]	Total sample of 3691 individuals with PKU 18,455 individuals without PKU as matched controls All individuals included in the MA	Individuals with PKU: 34.6 ± 14.3; range 20–NR Matched controls: 34.9 ± 14.2	Not explicitly stated but implied as early treatment after newborn diagnosis No information on diet adherence or Phe levels	Matched controls without PKU based on age, sex, geographic region, race, index-year, insurance type, and length of time of continuous enrollment in the database during follow-up	- Number of individuals with osteoporosis
Choukair et al. 2017 [38]	Total sample of 56 individuals with PKU All individuals included in the MA	26.0 ± 8.9; range, 11.8–41.5	Early treatment with a Phe-restricted diet (Phe-free) All PKU: mean blood Phe 605 SD ± 275 µmol/L; range 78–1326 Adults: mean blood Phe 642 SD ± 274 µmol/L; range 78–1326 Adolescents: mean blood Phe 361 SD ± 110 µmol/L; range 275–577	No explicit comparator BMD Z-score implicitly compares with a reference value based on healthy, age, and gender matched controls	- Mean BMD Z-scores: 1. Radius
de Groot et al. 2012 [39]	Total sample of 53 individuals with PKU Number of individuals included in the MA: 18 (individuals ≥ 20 years)	27.2 ± 4.9	Early treatment with a Phe-restricted diet (Phe-free) Treatment aims for all ages in individuals born prior to 2002: blood Phe concentration 200–500 µmol/L^b^ Treatment aims for individuals born in 2002 onwards: ≥ 12 years blood Phe concentration 120–600 µmol/L	No explicit comparator BMD Z-score implicitly compares with a reference value based on healthy, age, and gender matched controls	- Mean BMD Z-scores: 1. Lumbar spine
Lage et al. 2010 [16]	Total sample of 47 individuals with PKU Number of individuals included in the MA: 22 (Group 3: 19–42 years group)	29.3 ± 6.6; range, 19–42	Treatment with a Phe-restricted diet (Phe-free) Plasma Phe concentrations were used to evaluate dietary compliance: Target for individuals ≥ 18 years: < 600 µmol/L – *n* = 11/22 met the target Mean plasma Phe in Group 3 746.8 SD ± 280.4 µmol/L	No explicit comparator BMD Z-score implicitly compares with a reference value based on healthy, age, and gender matched controls	- Mean BMD Z-scores: 1. Lumbar spine 2. Femoral neck - Prevalence of Z-scores: 1. < -1.0 2. -1.0 to -2.5 3. ≤ -2.5 - Number of patients and %
Lubout et al. 2020 [10]	Total sample of 183 individuals with PKU All individuals included in the MA	Median: 32; range, 19–53	Early treatment with a Phe-restricted diet (Phe-free) Natural protein intake was categorized according to the following groups; (a) missing or not adherent to a diet – *n* = 16 (b) severe protein restriction (≤ 10 g/day) – *n* = 24 (c) moderate protein restriction (> 10–20 g/day) – *n* = 21 (d) mild protein restriction (> 20–40 g/day) – *n* = 16 (e) protein intake > recommended intake (> 40 g/day) – *n* = 23 Mean Phe concentration 775, range 61–1816 µmol/L	No explicit comparator BMD Z-score implicitly compares with a reference value based on healthy, age, and gender matched controls	- BMD Z-scores: 1. Lumbar spine: mean Z-score 2. Femoral neck: mean Z-score 3. Radius: mean Z-score 4. Total body: median Z-score - Prevalence of Z-scores: 1. ≤ -2.0 - Number of patients and %
Modan-Moses et al. 2007 [40]	Total sample of 31 individuals with PKU All individuals included in the MA	25 ± 5.3; range, 19–41	Early treatment with a Phe-restricted diet Individual dietary compliance was estimated from laboratory studies of Phe concentrations Mean Phe concentrations: All PKU: 968 SD 526 µmol/L Diet-adherent individuals: 823 SD 41 µmol/L Diet non-adherent individuals: 1192 SD 762 µmol/L Of 17 individuals who reported they were compliant, *n* = 8 (32%) had recommended Phe levels (< 726 µmol/L or < 12 mg/dL) Difference in mean Phe levels for diet-adherent versus diet non-adherent was not statistically significant	No explicit comparator BMD Z-score implicitly compares with a reference value based on healthy, age, and gender matched controls	- Mean BMD Z-scores: 1. Femoral neck 2. Total body - Prevalence of Z-scores: 1. -1.0 to -2.5 2. < -2.5 - Number of patients and %
Pérez-Dueñas et al. 2002 [19]	Total sample of 28 individuals with PKU Number of individuals included in the MA: 14	25.6; range, 19–33	Phe-restricted diet (Phe-free) 12 individuals were diagnosed with PKU after neonatal status (considered late treated) 2 individuals were diagnosed with PKU as neonates (considered early treated) 8 adults (57.1%) had good dietary compliance (Phe concentration ≤ 700 µmol/L) 6 adults (42.9%) had poor dietary compliance (Phe concentration > 700 µmol/L)	No explicit comparator BMD Z-score implicitly compares with a reference value based on healthy, age, and gender matched controls	- Individual BMD Z-scores: 1. Lumbar spine - Prevalence of BMD Z-scores: 1. < -1.0 2. -1.0 to -2.5 3. ≤ -2.0 4. ≤ 2.5
Stroup et al. 2017 [12]	Total sample of 8 individuals with PKU All individuals included in the MA	27.3; range, 16–35	Early treatment with a Phe-restricted diet (low-Phe diet with amino acid based or glycomacropeptide based medical food) Average blood Phe was not significantly different between treatments: Amino acid based medical food—401 SE 60 µmol/L Glycomacropeptide based medical food—469 SE 60 µmol/L	No explicit comparator BMD Z-score implicitly compares with a reference value based on healthy, age, and gender matched controls	- Individual BMD Z-scores: 1. Lumbar spine 2. Femoral neck 3. Radius 4. Total body - Prevalence of BMD Z-scores: 1. < -1.0 2. -1.0 to -2.5 3. ≤ -2.0 4. ≤ 2.5
Zeman et al. 1999 [41]	Total sample of 44 individuals with PKU All individuals included in the MA	16.1; range, 6–29	Early treatment with a Phe-restricted diet (Phe-free) Reported Phe levels were not explicit for all age groups but increased with age: 5 years – average 520 µmol/L 10 years – approximately 650 µmol/L 15 years – 900 µmol/L	No comparator BMD Z-score implicitly compares with a reference value based on child measurements of a healthy European pediatric population	- Prevalence of BMD Z-scores: 1. < -1.0 2. -1.0 to -2.5 3. ≤ 2.5 - Number of individuals and %

*BMD* Bone mineral density, *DXA* Bone density scan, *MA* Meta-analysis, *NR* Not reported, *Phe* Phenylalanine, *PKU* Phenylketonuria, *SD* Standard deviation, *SE* Standard error

^a^*P* value not reported

^b^No significant correlation (*p* = 0.159) between BMD Z-score and mean individual blood Phe (within treatment aim or above), no individual had a mean blood Phe below treatment aim in the year prior to DXA scanning, no significant correlation between BMD Z-score and number of times blood Phe below treatment aim in the year prior to DXA scanning (*p* = 0.699), proportion of blood Phe concentrations below reference range (*p* = 0.541), or mean cumulative variation of blood Phe concentration (*p* = 0.852), BMD Z-score was not significantly correlated to pre-treatment Phe concentration (*p* = 0.912) nor Phe tolerance at 24 months (*p* = 0.467)

### Study risk of *bias* assessment

**Fig. 2:**
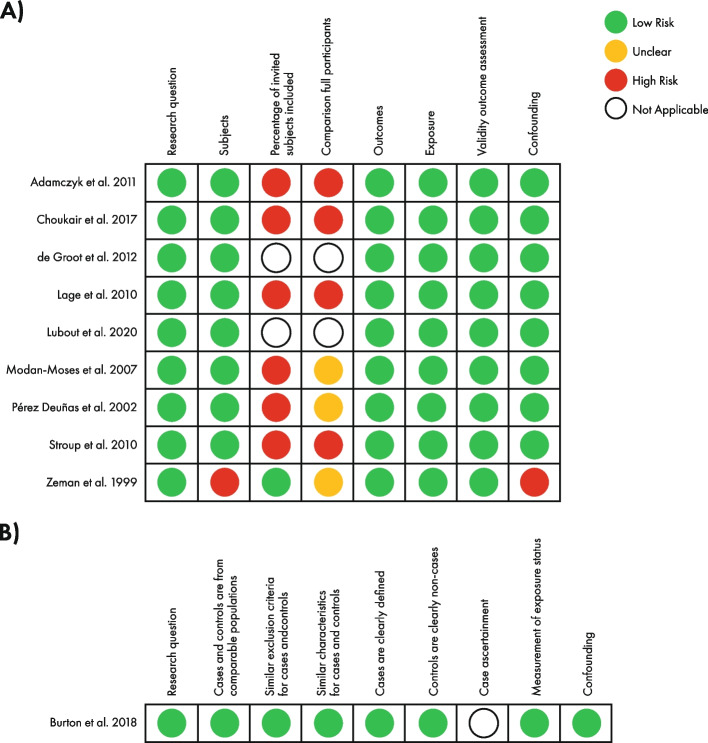
Risk of bias according to the SIGN checklist [42] for **A**) cohort studies and **B**) case–control studies. SIGN, Scottish Intercollegiate Guidelines Network

Out of nine studies assessed against the SIGN checklist for cohort studies, six studies [10, 12, 19, 38, 39, 41] were rated as being of acceptable quality; two [10, 39] of the six were retrospective in design and initially rated as high quality. The SIGN checklist, however, states that retrospective studies are only able to achieve “acceptable” quality at best and as such have been rated as acceptable given the SIGN guidelines. Three studies [16, 37, 40] were rated as high quality against the same checklist (Fig. 2A). The single study [8] assessed against the SIGN checklist for case–control studies was considered acceptable quality; again, despite being scored as high quality across some categories, a rating of acceptable quality was reached due to the study being retrospective (Fig. 2B). None of the ten studies was rated as being of unacceptable quality; therefore, no studies were excluded from the meta-analyses based on a rating of poor methodological quality.

### *Meta*-analysis of BMD in adults with PKU on a Phe-restricted diet versus a reference (non-PKU) population

#### BMD Z-scores for various bones

**Fig. 3:**
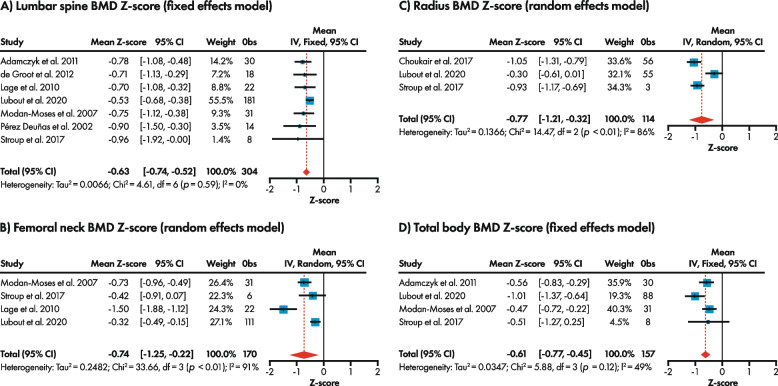
Forest plot of BMD Z-scores for adults with PKU on a Phe-restricted diet versus the respective reference (healthy) population (BMD Z-score = 0) for **A**) lumbar spine, **B** femoral neck, **C** radius, and **D** total body. BMD, bone mineral density; CI, confidence interval; df, degrees of freedom; I^2^, heterogeneity; IV, inverse variance; Obs, observations; Phe, phenylalanine; PKU, phenylketonuria. Effect size was estimated using either a fixed or random effects model based on the degree of heterogeneity in the studies included in the meta-analysis. Pooled mean Z-scores for each bone location were statistically significantly lower versus a reference (non-PKU) population

Seven studies [10, 12, 16, 19, 37, 38, 40] contributed to the meta-analysis of mean lumbar spine BMD Z-scores in individuals with PKU on a Phe-restricted diet (including those with uncontrolled, as well as those with controlled, blood Phe levels) versus a reference (non-PKU) population, where the Z-score is the number of standard deviations by which BMD differs from that expected for age and sex. The pooled mean BMD Z-score was -0.63 (95% CI: -0.74, -0.52), indicating that adults with PKU on a Phe-restricted diet have a statistically significantly lower mean lumbar spine BMD Z-score compared with a non-PKU population, but within the expected range for age according to ISCD OP criteria (> -2.0 [15]) (Fig. 3A). The pooled mean femoral neck BMD Z-score for those with PKU on a Phe-restricted diet was -0.74 (95% CI: -1.25, -0.22) [10, 12, 16, 40] and, as observed with lumbar spine, was statistically significantly lower than the non-PKU population but was still within the expected range for age (Fig. 3B). Meta-analysis of radius BMD Z-scores yielded a similar result: the pooled mean radius BMD Z-score [10, 12, 39] was statistically significantly lower than the non-PKU population but within the expected range for age (-0.77 [95% CI: -1.21, -0.32]) (Fig. 3C). The effect estimate for total body BMD Z-score reflected other bones, with a pooled mean total body BMD Z-score of -0.61 (95% CI: -0.77, -0.45) [10, 12, 37, 40] (Fig. 3D).

There was limited evidence of publication bias for lumbar spine BMD Z-scores, with a symmetrical scattering of points on the funnel plot (Additional file 2: Fig. S1A). Funnel plots of BMD Z-scores at all other bones assessed showed asymmetric scattering of points indicating potential publication bias (Additional file 2: Fig. S1B–D).

#### Sensitivity analysis

Repeating the analysis of BMD Z-scores but including only studies conducted exclusively in an adult population (≥ 18 years of age) yielded similar effect estimates to the overall analysis. A similar degree of heterogeneity to the overall analysis was also observed in this sensitivity analysis. Mean BMD Z-scores in adults ≥ 18 years of age with PKU on a Phe-restricted diet were -0.56 (95% CI: -0.68, -0.44) for lumbar spine, -0.83 (95% CI: -1.50, -0.17) for femoral neck, -0.62 (95% CI: -1.24, -0.01) for radius, and -0.64 (95% CI: -0.85, -0.44) for total body; these Z-scores were statistically significantly lower than those of the reference (non-PKU) population, but within the expected range for age, the same as was observed in the overall analysis.

#### Prevalence of low BMD Z-scores at pre-specified thresholds (any location)

**Fig. 4:**
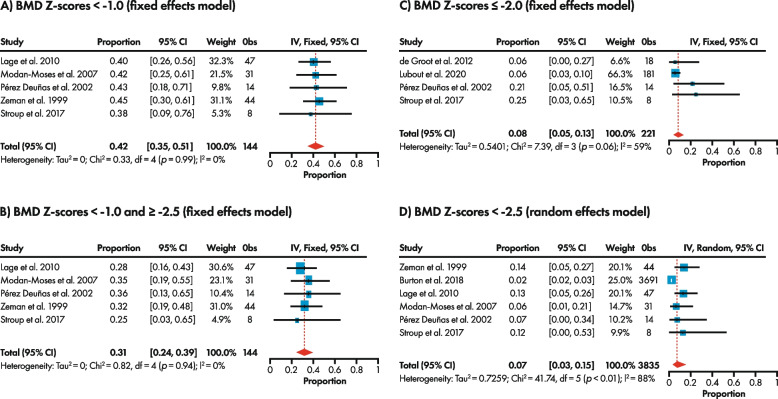
Forest plot for prevalence of BMD Z-score thresholds of: **A** < -1.0, **B** < -1.0 and ≥ -2.5, **C** ≤ -2.0, and **D** < -2.5, in adults with PKU on a Phe-restricted diet. BMD, bone mineral density; CI, confidence interval; df, degrees of freedom; I^2^, heterogeneity; IV, inverse variance; Obs, observations; Phe, phenylalanine; PKU, phenylketonuria. Effect size was estimated using either a fixed or random effects model based on the degree of heterogeneity in the studies included in the meta-analysis

An estimated 42% (95% CI: 35%, 51%) of those with PKU on a Phe-restricted diet [12, 16, 19, 40, 41] had BMD Z-scores < -1.0, a threshold indicating possible osteopenia or osteoporosis [19] (Fig. 4A). Considering low BMD Z-scores against a threshold indicative of osteopenia only (< -1.0 and ≥ -2.5) [16, 19], the prevalence was estimated at 31% (95% CI: 24%, 39%) (Fig. 4B).

A BMD Z-score of ≤ -2.0 is considered below that expected for age, according to the ISCD OP [15], and the prevalence of BMD Z-scores against this threshold was estimated at 8% (95% CI: 5%, 13%) [10, 12, 19, 39] (Fig. 4C). The lowest threshold evaluated was a BMD Z-score of < -2.5, indicating a secondary cause of osteoporosis [16, 17], with a prevalence of 7% (95% CI: 3%, 15%) [8, 12, 16, 19, 40, 41] (Fig. 4D).

##### Sensitivity analysis

To note, one study, Burton et al. [8], reported the prevalence ratio (rate per 100 person years based on health insurance claims data, adjusted for participant baseline characteristics) of osteoporosis only and not BMD Z-scores explicitly. For the purposes of the meta-analysis, it was assumed that those individuals in the study with a diagnosis of osteoporosis would have a BMD Z-score of < -2.5 [16–18]. A sensitivity analysis was conducted in which the Burton et al. study was removed, with little impact on the effect estimate.

Publication bias was not considered a concern across the BMD Z-score < -1.0, BMD Z-score < -1.0 and ≥ -2.5, and BMD Z-score ≤ -2.0 prevalence thresholds, as funnel plots showed the scattering of points was reasonably symmetric. The funnel plot of prevalence of BMD Z-scores < -2.5 showed asymmetric scattering of points, suggesting publication bias may be a concern (Additional file 3: Fig. S2).

#### Subgroup analysis of lumbar spine BMD Z-scores

For the analysis of lumbar spine BMD Z-scores by decade of study publication (Additional file 4. Fig. S3A), there was one study [41] that was conducted in 1999 but only reported data for the secondary objective; therefore, this study was not considered in this particular subgroup analysis. All other studies were conducted between 2001 and 2020 and were included in the subgroup analysis by decade of study publication (2001–2010 and 2011–2020). Mean lumbar spine BMD Z-scores by decade of study publication (2001–2010 or 2011–2020) in individuals with PKU on a Phe-restricted diet were not significantly lower than mean lumbar spine BMD Z-scores of the respective general population (Additional file 4. Fig. S3A). There was no significant difference in mean BMD Z-scores between the subgroups (*p* = 0.09*;* Table 3; Additional file 4: Fig. S3A). Mean femoral neck BMD Z-scores and mean total body BMD Z-scores in those with PKU on a Phe-restricted diet, by decade of study publication, were also statistically significantly lower than those from the reference (non-PKU) population (Additional file 5: Table S2). Statistically significant differences between the decade of study publication subgroups were observed for femoral neck and total body, albeit with a differing direction of effect for each bone location (higher BMD Z-score in the 2011–2020 subgroup for femoral neck, and higher BMD Z-score in the 2001–2010 subgroup for total body); however, the 95% CIs of the decade subgroups overlap indicating potential statistical insignificance (Additional file 5: Table S2).

**Table 3:** Lumbar spine BMD Z-scores for adults with PKU on a Phe-restricted diet versus a healthy population (BMD Z-score = 0): subgroup analysis

Subgroup		Total sample per subgroup	Mean BMD Z-score (95% CI)	Heterogeneity (I^2^ test)	Subgroup difference, chi-square test
**Decade of study publication**^**a**^	**2001–2010**^**b**^ [16, 19, 37, 40]	97	-0.76 (-0.95, -0.58)^d^	0%^e^	*p* = 0.09
	**2011–2020 ** [10, 12, 39]	207	-0.56 (-0.70, -0.42)^d^		
**Blood Phe level control**^**c**^	**Controlled ** [19, 37]	26	-0.40 (-0.77, -0.03)^d^	78%^f^	*p* < 0.01
	**Uncontrolled ** [19, 37]	18	-1.22 (-1.73, -0.72)^d^		
**Gender**	**Male ** [12, 19]	7	-1.71 (-2.86, -0.57)^d^		
	**Female ** [12, 19]	15	-0.58 (-1.05, -0.11)^d^	24%^e^	*p* = 0.07

*BMD* Bone mineral density, *CI* Confidence interval, *Phe* Phenylalanine, *PKU* Phenylketonuria

^a^Analysis by decade of publication of studies was conducted as a proxy for reported improvements in Phe-restricted dietary supplements over time

^b^Adamczyk et al. [37] was included in the 2001–2010 decade of publication because it was published online in August 2010, although it was not published in print until 2011

^c^Adherence to diet was defined by blood Phe level; each study used a different blood Phe threshold to define adherence

^d^Statistically significantly lower mean Z-score versus a reference (healthy) population

^e^Effect size was estimated using a fixed effects model based on the overall level heterogeneity score (considering both subgroups) [32]

^f^Effect size was estimated using a random effects model based on the overall level heterogeneity score (considering both subgroups) [32]

For the analysis of lumbar spine BMD Z-scores by PKU population whose blood Phe levels were controlled versus uncontrolled (used as a proxy for adherent versus non-adherent to a Phe-restricted diet; Table 3; Additional file 4. Fig. S3B), there were two studies that could not be included: one study [40] was excluded because adherence was self-reported and not directly related to blood Phe-level measurements, and the other study [39] was excluded because sample sizes for the blood Phe-level controlled versus uncontrolled populations were not reported. Mean lumbar spine BMD Z-scores, both in those with PKU whose blood Phe levels were controlled and in those whose blood Phe levels were uncontrolled, were significantly lower than the mean lumbar spine BMD Z-scores from a reference (non-PKU) population (Table 3; Additional file 4: Fig. S3B). When comparing the mean lumbar spine BMD Z-scores between the two subgroups (controlled versus uncontrolled), the results of the chi-square test indicate that the difference was statistically significant (*p* < 0.01) (Table 3; Additional file 4: Fig. S3B); however, the overlapping 95% CIs between the two subgroups conflicts with this result, suggesting that the difference may not be significant.

Mean lumbar spine BMD Z-scores in females and males with PKU were significantly lower than those in the reference (non-PKU) population (Table 3; Additional file 4: Fig. S3C). The difference in mean lumbar spine BMD Z-scores between the two subgroups (male vs. female) was approaching statistical significance (*p* = 0.07; Table 3; Additional file 4: Fig. S3C).

## Discussion

### Quality of evidence

The studies included in the meta-analysis were identified as part of a broader SLR evaluating somatic comorbidities and can be considered the best available evidence. All studies had a rating of at least acceptable quality when assessed by SIGN checklists. No studies were excluded from the meta-analysis because they were considered unacceptable quality. The small number of studies with BMD Z-scores for femoral neck, radius, and total body prevented a reliable assessment of publication bias. However, for all other analyses, there was limited or no evidence of publication bias.

### Main findings and relation to prior research

#### BMD Z-scores for various bones

The results of this meta-analysis suggest that adults with PKU on a Phe-restricted diet have statistically significantly lower mean BMD Z-scores for various bones (and total body) compared with the respective reference (non-PKU) population (where the Z-score is the number of standard deviations by which BMD differs from that expected for age and sex); however, the pooled BMD Z-scores are still within the expected range for age (> -2.0) [15].

This outcome aligns with the published findings in Demirdas et al. [9] (Table 4), who performed a meta-analysis of BMD Z-scores for various bones in a PKU population spanning a broader age group (including children). In addition, Rojas-Agurto et al. [43] measured BMD in young adults (aged 19–27 years) with PKU. Although this study was included in the broader SLR, it did not meet criteria for inclusion in the meta-analysis because BMD in g/cm^2^, rather than Z-score, was reported. Two groups of individuals with PKU were included in the study: group 1 included those who used a protein substitute without Phe; group 2 included those who used a protein substitute without Phe up to the age of 18 years and then followed a mostly vegan diet. Both groups were matched by age and sex with non-PKU controls. The study found a significant reduction in spine and femoral BMD when compared with non-PKU controls. However, while BMD was found to be lower than the non-PKU population reference range, the BMD for this sample was interpreted as clinically normal, in line with our meta-analysis results and those published by Demirdas et al. [9].

**Table 4:** Comparison of mean BMD Z-scores with published data [9]

**Bone location**	**Results from meta-analysis**		**Results from Demirdas et al. 2015 ** [9]	
	**Sample size**^**a**^	**Mean (95% CI)**	**Sample size**^**b**^	**Mean (95% CI)**
**Lumbar spine**	309	-0.59 (-0.70, -0.48)	247	-0.70 (-0.82, -0.57)
**Femoral neck**	170	-0.77 (-1.33, -0.21)	78	-0.96 (-1.42, -0.49)
**Total body**	157	-0.61 (-0.77, -0.45)	131	-0.45 (-0.61, -0.28)

*BMD* Bone mineral density, *CI* Confidence interval, *PKU* Phenylketonuria

^a^Includes adults with PKU as defined by each individual study. For some, this means the individual reached sexual maturity

^b^Includes individuals of any age

Although our meta-analysis may have included data from studies in which some individuals were < 18 years of age (and therefore not strictly considered as being of adult age), repeating the analysis using data from studies conducted exclusively in an adult population (≥ 18 years of age) yielded a similar outcome, suggesting that the findings of lower mean BMD Z-scores are robust and can be regarded with a high level of certainty. This meta-analysis adds weight to the already published evidence on low mean BMD Z-scores in individuals with PKU on a Phe-restricted diet [9, 21], benefitting from a larger sample size (vs. Demirdas et al. [9]) for various bones, and with a specific focus on adults. In particular, the inclusion of Lubout et al. [10] contributed a large sample of adults with PKU to each bone category (181, 111, and 88 adults for lumbar spine, femoral neck, and total body, respectively) thereby reducing some of the heterogeneity and uncertainty associated with the results, particularly for lumbar spine.

The impact of different population and study characteristics on mean BMD Z-scores was explored by subgroup analyses, but a high degree of uncertainty in the results has limited interpretation. Exploring whether improvements to diet over time may be positively impacting bone health, the pooled mean lumbar spine BMD Z-score was higher in adults with PKU on a Phe-restricted diet from studies published in 2011–2020 versus those studies published in 2001–2010; however, the difference was not statistically significant. Although statistically significant differences between subgroups (2001–2010 and 2011–2020) were observed for pooled mean femoral neck and total body BMD Z-scores, the direction of effect for each location was different and overlapping of the 95% CIs indicates uncertainty associated with the results. Therefore, it was not possible to draw conclusions on whether improvements to MNT (i.e. the Phe-restricted diet) over time have had a direct impact on bone health.

An attempt was made to explore the impact of adherence to the Phe-restricted diet (as assessed by controlled versus uncontrolled blood Phe levels). Lumbar spine BMD Z-scores in diet-adherent and non-adherent populations were analyzed, where adherence to diet was defined by blood Phe level (i.e. low blood Phe versus high blood Phe). Only two studies [19, 37] contributed to the analysis, each using different blood Phe level thresholds to define adherence. The estimate of effect is uncertain; however, pooled mean lumbar spine BMD Z-scores were higher in adults with low blood Phe levels than in those with high blood Phe levels. A positive correlation between blood Phe level and spontaneous osteoclastogenesis in those with PKU has been reported in the literature [44], meaning a high blood Phe level may lead to low BMD through increased osteoclast activity and bone resorption, a hypothesis that other researchers have also highlighted [16, 44]. However, two studies investigating the impact of dietary adherence or blood Phe level control on BMD, which could not be included in the meta-analysis, did not find a statistically significant impact [39, 40]. Moden-Moses et al. [40] reported lower lumbar, femoral neck, and total body BMD Z-scores in individuals who self-reported dietary compliance compared with those who reported non-compliance, but the difference between groups was not statistically significant for BMD Z-scores nor mean blood Phe levels. Interestingly, of the 17 individuals self-reporting dietary compliance, only eight had recommended blood Phe levels. Furthermore, de Groot et al. [39] found no significant correlation between BMD Z-score and: mean individual blood Phe level (within treatment aim or above), number of times blood Phe level was below treatment aim in the year prior to bone density scanning, proportion of blood Phe concentrations below reference range, mean cumulative variation of blood Phe concentration, pre-treatment Phe concentration, or Phe tolerance at 24 months of age.

It is important to note that non-adherence to a Phe-restricted diet may not be the sole reason why blood Phe levels are high. For example, elevated blood Phe levels may be due to low Phe tolerance linked to increased disease severity (and in turn, linked to mutation status and level of functional PAH) [5, 6]; lack of access to MNT, which varies by country, may also impact patient compliance. Therefore, it is perhaps appropriate to question the extent to which blood Phe levels can proxy for adherence.

Analysis of mean lumbar spine BMD Z-scores by gender revealed a considerably lower pooled mean score in males versus females; however, the difference was not statistically significant and only two studies [19, 37] contributed to the analysis, both with small sample sizes, limiting interpretation. Nevertheless, this finding is noteworthy since males, in general, have a higher BMD compared with females [45]. Another study has reported a similar finding in individuals with PKU (lower mean BMD Z-score reported in males compared with females) [46]. This study was not indexed as a human study and so was not identified by the literature search conducted for the SLR but was identified from subsequent horizon scanning. As this study did not present results separately for individuals with PKU on a Phe-restricted diet alone, and instead grouped these results together with those from individuals also receiving tetrahydrobiopterin, this study was not eligible for inclusion in the meta-analysis.

#### Prevalence of low BMD Z-scores at pre-specified thresholds

Despite pooled mean BMD Z-scores falling within the expected range for age (> -2.0), a large proportion (42%) of adults with PKU had BMD Z-scores that passed the threshold indicating possible osteopenia or osteoporosis (< -1.0), and 32% had BMD Z-scores that passed the threshold indicating possible osteopenia only (< -1.0 and ≥ -2.5).

In this meta-analysis, fewer than 1 in 10 adults with PKU (8%) had a BMD Z-score below the expected range for age (≤ -2.0). Previously, Demirdas et al. [9] investigated the prevalence of BMD Z-scores and showed that approximately 10% of those with early treated PKU are expected to have low BMD Z-scores (< -2.0) given their age; therefore, the results reported in this meta-analysis are in line with the estimates provided by Demirdas and colleagues [9]. In addition, Demirdas et al. compared their prevalence of low BMD Z-scores estimate in the PKU population with an estimated prevalence of low BMD Z-scores in the general population [9]. Using the National Health and Nutrition Examination Survey dataset, Demirdas et al. estimated the prevalence of BMD Z-score < -2.0 to be 2.3% in the general population, which is lower than the estimated prevalence of BMD Z-score < -2.0 in individuals with PKU, both in this study and in Demirdas et al. (8% and 10%, respectively).

### Clinical implications

Abnormal bone status, including as a possible consequence of dietary treatment, has been a concern for a long time in patients with PKU [4, 9]. Low BMD can lead to severe outcomes, including osteopenia and osteoporosis, and eventually fracture [13], which is associated with high medical costs [47].

The most important finding of the meta-analysis is that the proportion of patients with low BMD is higher in the PKU population than in the non-PKU (healthy or general) population. However, the meta-analysis was not designed to fully investigate if the impact of the Phe-restricted diet on bone health status, as measured by BMD Z-scores, is distinct from the impact of PKU. In addition, given the lack of consensus on how to classify low BMD Z-scores, we cannot draw firm conclusions on the clinical implications of the results reported in this meta-analysis.

### Strengths and limitations

The meta-analysis included studies that had been identified as part of a broader SLR, the strengths and limitations of which have been documented in full in a separate publication. To note, the SLR search scope was broad, incorporating terms for aspects of bone health including BMD, bone mineral content, osteoporosis, and bone loss; however, osteopenia was not included, meaning some relevant records may have been missed.

The meta-analysis focused on an adult population (individuals aged ≥ 16 years or classified as sexually mature) with PKU and on a Phe-restricted diet, and the findings reported build on existing published BMD data in mixed-age populations [9, 21]. A minor limitation of the meta-analysis of BMD Z-scores was the inability to exclude all individuals with PKU who were < 16 years of age [37, 38] because the exact proportions of these individuals in two studies could not be ascertained [37, 38]. However, the number of individuals < 16 years of age in these studies was very low (mean age 17.3 (± 2.2) [37] and 26.0 (± 8.9) [38]). Furthermore, removal of studies including data from individuals < 18 years of age [12, 37, 38] in a sensitivity analysis yielded similar effect estimates and had a similar degree of heterogeneity, to the overall analysis; hence, the inclusion of data related to individuals < 16 years of age was unlikely to have had a major impact on the results. Similarly, it was not possible to exclude these data from two other studies [16, 41] included in the meta-analysis of the prevalence of low BMD Z-scores because they reported only the percentage of individuals in their sample who had BMD Z-scores within defined ranges.

Z-score was chosen as the most relevant effect measure to use in the meta-analysis, as it compares bone density in an individual to that of an age and gender matched control and is always used to assess BMD in children, young adults, pre-menopausal women, and men < 50 years of age [48]; most studies reported Z-scores rather than alternative measures, such as T-score or g/cm^3^, and most individuals in the included studies were relatively young. However, it is encouraged that future studies report multiple but consistent measures of BMD.

A lack of standardization of the definitions of low BMD makes interpretation of the clinical relevance of the data challenging, where a BMD Z-score > -2.0 is considered to be within the expected range for age [15], yet a BMD Z-score < -1.0 is considered to be indicative of osteopenia [16, 19]. A consensus regarding BMD Z-scores is encouraged and may help to promote collection of standardized, BMD outcome measures that are consistent across clinical studies of individuals with PKU, yielding more robust results when combined via meta-analysis.

This study did not consider fracture history as part of the analysis of bone health in adults with PKU on a Phe-restricted diet. The ISCD OP states that in some populations (i.e. children and adolescents, males under the age of 50 years) BMD Z-scores alone cannot be used as an indicator of osteoporosis [15, 20]; for example, in children and adolescents, both a clinically significant fracture history and a BMD Z-score ≤ -2.0 are needed [20].

The individuals with PKU included in this study were mostly early treated (Table 1); therefore, it was not possible to determine the impact of delayed metabolic control on long-term bone health. Limited data were available to investigate the impact of adequate blood Phe-level control on BMD and the lack of standardization in recommended blood Phe levels in adults between studies (2–10 mg/dL in those aged ≥ 12 years [37] versus < 700 µmol/L [~ 8 mg/dL] [19]) limits interpretation. This meta-analysis did not consider whether individuals were on treatment for specific bone-related conditions (e.g. osteoporosis) and so could not determine the potential impact of treatments on bone health over time. Investigating the connection between BMD and Phe-restricted diet composition was out-of-scope, and as discussed earlier, using blood Phe level as proxy for adherence may not be appropriate.

Other limitations of the data include heterogeneity in the results of the primary objective analysis, and associated subgroup analyses (with small sample sizes), leading to uncertainty in the findings, limiting interpretation. Insufficient homogenous data prevented planned subgroup analyses by severity of PKU (HPA, mild PKU, moderate PKU, classical PKU) and by risk of bias.

### Future studies

This meta-analysis highlights the need for additional studies using similar outcome measures and thresholds for low BMD to reduce uncertainty in the findings published to date, and to permit new analyses in other specific subgroups (e.g. by PKU disease severity, early versus late-treated, individuals treated for osteopenia or osteoporosis). A network meta-analysis of individuals with PKU on different therapies (given sufficient available evidence) may also aid understanding of the dietary and/or pharmacologic treatment effect on BMD.

Further research is warranted to understand the holistic impact of PKU on bone health and the relationship between effective metabolic control (by dietary and/or pharmacologic intervention) and maintenance of bone health, including the effect of differences in blood Phe levels considered ‘effective’ metabolic control, on comorbidities in adults with PKU, including bone health, to facilitate global agreement on recommended blood Phe levels. Lack of consensus complicates analysis of the impact of dietary adherence.

At present, the predominant cause of low BMD in the PKU population is unclear [10]. There are multiple factors that may influence BMD, such as degree of physical activity; inadequate nutritional intake from protein substitutes [49, 50] and the influence of amino acid supplementation on mineral excretion [11, 12, 51], dependence of vitamin D levels on the intake of protein substitutes [52] and the potential impact of defective vitamin D metabolism, which may impact skeletal health [53]; non-adherence to MNT [54, 55] and/or inadequate management of pharmacologic therapy [56]. Therefore, studies should be designed to isolate the impact of a Phe-restricted diet on BMD, as well as to investigate how adherence/compliance with medical interventions (whether dietary and/or pharmacologic) affects nutritional intake and thereby markers of bone health. It would also be beneficial to design studies to understand whether improvements in MNT over time have reduced the impact of the Phe-restricted diet on BMD and resulted in improved bone health in individuals with PKU.

Given the time period covered by the studies included in our analysis, it would be interesting to compare the protein substitutes that study participants were receiving, to explore whether the nutritional quality of the Phe-restricted diets varied between older and more recent studies. In the absence of patient-level data, the reimbursement status of protein substitutes in the countries where data were collected and analyzed could be examined; however, this may be difficult to interpret as full reimbursement is not a guarantee of product prescription nor patient compliance.

A consideration of fractures in adults with PKU on a Phe-restricted diet, as a possible sequela of low BMD, was not part of this study; however, the incidence of fracture warrants investigation, including the relationship with other factors such as age, sex, treatment, and BMD, as well as the associated economic burden.

## Conclusions

Adults with PKU on a Phe-restricted diet have lower mean BMD Z-scores than a non-PKU population but are still generally within what is considered the expected range for age (> -2.0). In this study, fewer than 1 in 10 adults with PKU had a BMD Z-score below the expected range for age (≤ -2.0), although more than one-third (42%) of adults with PKU had a BMD Z-score passing the threshold that is considered indicative of possible osteopenia or osteoporosis (< -1.0). Further studies are needed to confirm the clinical implications of a low, but within expected range, BMD Z-score, including any increased risk of fracture.

The low numbers of studies evaluating BMD Z-scores in adults with PKU precludes a robust analysis of which population characteristics are most impacting BMD; the role of the Phe-restricted diet, and differences in recommended blood Phe levels, on BMD, as well as any additional impact from the disease itself.

## Supplementary Information

Additional file 1.

Additional file 2.

Additional file 3.

Additional file 4.

Additional file 5.

## Data Availability

The datasets, systematic literature review study protocol, and meta-analysis statistical analysis plan used and/or analyzed during the current study are available on reasonable request. Additional supporting documents may be available upon request. Investigators will be able to request access to these data and supporting documents via a data sharing portal (https://www.biomarin.com/our-science/funding-and-support/publication-data-request/) beginning 6 months and ending 2 years after publication. Data associated with any ongoing development program will be made available within 6 months after approval of the relevant product. Requests must include a research proposal clarifying how the data will be used, including proposed analysis methodology. Research proposals will be evaluated relative to publicly available criteria available at https://www.biomarin.com/our-science/funding-and-support/publication-data-request/ to determine if access will be given, contingent upon execution of a data access agreement with BioMarin Pharmaceutical Inc.

## Abbreviations

AA-MF: Amino acid medical foods
BMD: Bone mineral density
CI: Confidence interval
DXA: Bone density scan
F: Female
GMP-MF: Glycomacropeptide medical foods
HPA: Hyperphenylalaninemia
ISCD OP: International Society for Clinical Densitometry Official Position
M: Male
MD: Mean difference
MeSH: Medical Subject Headings
MNT: Medical nutrition therapy
NR: Not reported
PAH: Phenylalanine hydroxylase
Phe: Phenylalanine
PICOS: Population, Intervention, Comparator, Outcomes, Study designs
PKU: Phenylketonuria
PRISMA: Preferred Reporting Items for Systematic Reviews and Meta-Analyses
PUFA: Polyunsaturated fatty acid
SD: Standard deviation
SE: Standard error
SIGN: Scottish Intercollegiate Guidelines Network
SMD: Standardized mean difference
SLR: Systematic literature review
