# Supplementary material for: Meta-analysis of bone mineral density in adults with phenylketonuria
Source: Orphanet J Rare Dis. 2024 Sep 12;19:338. doi: 10.1186/s13023-024-03223-9 (PMC11391789; doi:10.1186/s13023-024-03223-9)
Supplement: Supplementary file 1 — Additional file 1. [file 13023_2024_3223_MOESM1_ESM.docx]

Meta-analysis of bone mineral density in adults with phenylketonuria

Júlio C. Rocha, Álvaro Hermida, Cheryl J. Jones, Yunchou Wu, Gillian E. Clague, Sarah Rose, Kaleigh B. Whitehall, Kirsten K. Ahring, André L.S. Pessoa, Cary O. Harding, Fran Rohr, Anita Inwood, Nicola Longo, Ania C. Muntau, Serap Sivri, François Maillot

# Supplementary information

Additional file 1: Table S1 PubMed^®^ search string

| Search no. | Query | Details | No. of hits |
| --- | --- | --- | --- |
| 1 | Disease | “Phenylketonuria”[Title/Abstract] OR “phenylketonuria*”[Title/Abstract] OR “phenylalanine hydroxylase deficien*”[Title/Abstract] OR “phenylalanine deficien*”[Title/Abstract] OR “phenylalanine deficiency syndrome”[Title/Abstract] OR “hyperphenylalanin*”[Title/Abstract] OR “hyper-phenylalanin*”[Title/Abstract] OR “PKU”[Title/Abstract] |  |
| 2 | Disease burden/ comorbidities | "Disease Burden"[Title/Abstract] OR “burden of disease”[Title/Abstract] OR "comorbid*"[Title/Abstract] OR “co-morbid*”[Title/Abstract] OR "complication*”[Title/Abstract] OR “Burden of illness”[Title/Abstract] OR “Illness Burden”[Title/Abstract] OR “somatic comorbid*”[Title/Abstract] OR “somatic co-morbid*”[Title/Abstract] |  |
| 4 | HRQoL burden | "quality of life”[MeSH Terms] OR “health-related quality of life"[Text Word] OR “health related quality of life”[Text Word] OR "HRQOL”[Text Word] OR “quality of life"[Text Word] OR "Life quality”[Text Word] OR “health status”[Text Word] OR "Sickness Impact Profile”[MeSH Terms] OR “Activities of Daily Living"[MeSH Terms] OR “medical outcomes survey”[Text Word] OR "disabilit*"[Text Word] OR “short form 36”[Text Word] OR "SF-36"[Text Word] OR “EQ-5D”[Text Word] OR “social isolation”[MeSH Terms] OR “social exclusion*”[Text Word] OR “ostracis*”[Text Word] OR “social impact”[Text Word] OR “social behavior”[Text Word] OR “social behaviour”[Text Word] OR “socio-occupational”[Text Word] OR “socio occupational” [Text Word] OR “social function*”[Text Word] OR “Stress Disorders, Traumatic, Acute”[MeSH Terms] OR “stress”[Title/Abstract] OR “stress”[Text Word] “chronic stress”[Text Word] OR “Oxidative stress”[MeSH Terms] |  |
| 5 | Bone density | (“bone density”[Title/Abstract]) OR (“bone mineral density”[Title/Abstract]) OR (“Bone Density”[MeSH Terms]) OR (“bone mineral densit*”[Text Word]) OR (“bone mineral content*”[Text Word]) OR (“Osteoporosis”[MeSH Terms]) OR (“post-traumatic osteoporos*”[Text Word]) OR (“chronic osteoporosis”[Text Word]) OR (“bone loss”[Text Word]) OR (“bone-loss”[Text Word]) |  |
| 6 | Cardiovascular disease | (“Risk of cardiovascular”[Title/Abstract]) OR (“Cardiovascular*”[Title/Abstract]) (Heart Diseases[MeSH Terms]) OR (Heart Disease*[Text Word]) OR (Cardiac Disease*[Text Word]) OR (Cardiac Disorder*[Text Word]) OR (Heart Disorder*[Text Word]) OR (Myocardial Infarction[MeSH Terms]) OR (Myocardial Infarction*[Text Word]) OR (heart attack*[Text Word]) OR (“Myocardial Infarct”[Text Word]) OR (Heart Failure[MeSH Terms]) OR (“heart failure”[Text Word]) OR (“Cardiac Failure”[Text Word]) OR (“Myocardial Failure”[Text Word]) OR (Cardiomyopathies[MeSH Terms]) OR (Cardiomyopath*[Text Word]) OR (myocardial disease*[Text Word]) OR (cardiac complication*[Text Word]) OR (heart complication*[Text Word]) OR (myocardial complication*[Text Word]) |  |
| 7 | Gastrointestinal disorders | (“Gastrointestinal”[Title/Abstract]) (“Gastro-intestinal”[Title/Abstract]) OR (Gastrointestinal Diseases[MeSH Terms]) OR (gastrointestinal disorder*[Text Word]) (gastro-intestinal disorder*[Text Word]) OR (functional gastrointestinal disorder*[Text Word]) OR (functional gastro-intestinal disorder*[Text Word]) |  |
| 8 | Dermatologic disorders | (“Dermatologic*”[Title/Abstract]) OR (Skin Diseases[MeSH terms]) OR (Atopic dermatitis[Text Word]) OR (Dermatitis[Text Word]) (skin tissue disorder*[Text Word]) OR (subcutaneous tissue disorder*[Text Word]) |  |
| 9 | Obesity/ overweight | (“obes*”[Title/Abstract]) OR (“overweight”[Title/Abstract]) OR (“BMI”[Title/Abstract]) OR (“body mass index”[Title/Abstract]) OR (Obesity[MeSH Terms]) OR (body weight[Text Word]) OR (Overweight[MeSH Terms]) |  |
| 10 | Diabetes | (“Diabet*”[Title/Abstract]) OR (“Insulin”[Title/Abstract]) OR (Diabetes Mellitus [MeSH Terms]) OR (anti diabet*[Text Word]) OR (anti-diabet*[Text Word]) |  |
| 11 | Migraine/ headache | (“Migraine*”[Title/Abstract]) OR (“headache*”[Title/Abstract]) OR (Headache Disorders[MeSH Terms]) OR (Migraine Disorders[MeSH Terms]) OR (headache*[Text Word]) OR (migraine[Text Word]) |  |
| 12 | COPD/asthma | (“COPD”[Title/Abstract]) OR (“chronic obstructive pulmonary disease”[Title/Abstract]) OR (“pulmonary disease*”[Title/Abstract]) OR (“lung disease”[Title/Abstract]) OR (“asthma”[Title/Abstract]) OR (Pulmonary Disease, Chronic Obstructive[MeSH Terms]) OR (Chronic Obstructive Lung Disease*[Text Word]) OR (Chronic Obstructive Pulmonary Disease*[Text Word]) OR (air flow obstruction*[Text Word]) OR (Chronic airflow obstruction*[Text Word]) OR (COPD[Text Word]) OR (COAD[Text Word]) OR (Asthma[MeSH Terms]) OR (asthma*[Text Word]) OR (bronchial asthma*[Text Word]) OR (chronic obstructive airway disease[Text Word]) |  |
| 13 | Cancer | (“cancer”[Title/Abstract]) OR (“neoplasm*”[Title/Abstract]) OR (Neoplasms[MeSH Terms) OR (neoplasia*[Text Word]) OR (Neoplasm*[Text Word]) OR (Tumor*[Text Word]) OR (Tumour[Text Word]) OR (cancer*[Text Word]) OR (malignanc*[Text Word]) |  |
| 14 | Neurologic disorders | (“neurologic*”[Title/Abstract]) OR (Nervous System Diseases[MeSH Terms) OR (Neurologic Disorder*[Text Word]) OR (Neurological Disorder*[Text Word]) OR (Nervous System Disorder*[Text Word]) OR (“tremor”[Text Word]) |  |
| 15 | Sleep disorders | (“Sleep Wake Disorders”[MeSH Terms]) OR (“sleep disorder*”[Text Word]) OR (“neurogenic tachypnea”[Text Word]) OR (“sleeper syndrome”[Text Word]) OR (“short sleep”[Text Word]) OR (“sleep disturb*”[Text Word]) OR (“insomnia”[Text Word]) OR (“wakefulness”[Text Word]) OR (“disturbed sleep”[Text Word]) OR (“sleep”[Text Word]) OR (“sleep pattern”[Text Word]) |  |
| 16 | Nutritional disorders | (“feeding and eating disorders”[MeSH Terms]) OR (“abnormal eating”[Text Word]) OR (“eating disorder”[Text Word]) OR (“nutritional disorder*”[Text Word]) OR (“nutrient deficienc*”[Text Word]) OR (“nutritional status”[Text Word]) OR (“protein consumption”[Text Word]) OR (“protein tolerance”[Text Word]) OR (“protein intolerance”[Text Word]) OR (“nutritional deficienc*”[Text Word]) OR (“diet*”[Text Word]) OR (“dietary composition”[Text Word]) OR (“disordered eating”[Text Word]) |  |
| 17 |  | 1 AND (2 OR 3 OR 4 OR 5 OR 6 OR 7 OR 8 OR 9 OR 10 OR 11 OR 12 OR 13 OR 14 OR 15 OR 16) | 6479 |
| 18 |  | (“Editorial”[Publication Type] OR “Letter”[Publication Type] OR “Comment”[Publication Type]) |  |
| 19 | Exclude unwanted study types | 17 NOT 18 | 6239 |
| 20 | Limit to studies on adults | 19 AND adult age filters | 1256 |
| 21 | Limit to human studies published in English | 20 AND human studies filter AND English language filter | 1128 |

Results column shows results from the original search conducted on February 1, 2022.

COAD, chronic obstructive airway disease; COPD, chronic obstructive pulmonary disease; HRQoL, health-related quality of life; MeSH, medical subject headings; PKU, phenylketonuria.
