# Supplementary material for: Meta-analysis of bone mineral density in adults with phenylketonuria
Source: Orphanet J Rare Dis. 2024 Sep 12;19:338. doi: 10.1186/s13023-024-03223-9 (PMC11391789; doi:10.1186/s13023-024-03223-9)
Supplement: Supplementary file 2 — Additional file 2. [file 13023_2024_3223_MOESM2_ESM.docx]

Meta-analysis of bone mineral density in adults with phenylketonuria

Júlio C. Rocha, Álvaro Hermida, Cheryl J. Jones, Yunchou Wu, Gillian E. Clague, Sarah Rose, Kaleigh B. Whitehall, Kirsten K. Ahring, André L.S. Pessoa, Cary O. Harding, Fran Rohr, Anita Inwood, Nicola Longo, Ania C. Muntau, Serap Sivri, François Maillot

# Supplementary information

Additional file 2: Fig. S1 Funnel plot of BMD Z-scores for adults with PKU on a Phe-restricted diet versus the respective reference (non-PKU) population for A) lumbar spine, B) femoral neck, C) radius, and D) total body


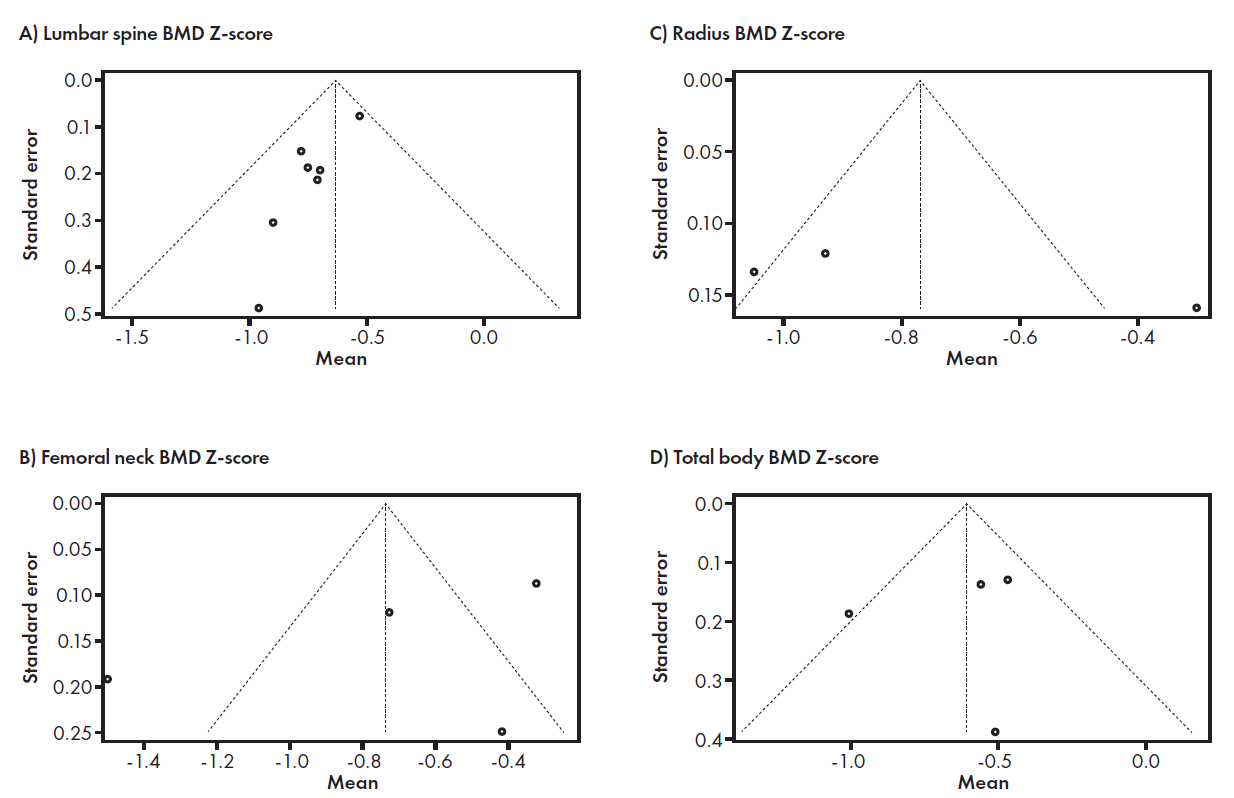


BMD, bone mineral density; Phe, phenylalanine; PKU, phenylketonuria.
