# Supplementary material for: Meta-analysis of bone mineral density in adults with phenylketonuria
Source: Orphanet J Rare Dis. 2024 Sep 12;19:338. doi: 10.1186/s13023-024-03223-9 (PMC11391789; doi:10.1186/s13023-024-03223-9)
Supplement: Supplementary file 3 — Additional file 3. [file 13023_2024_3223_MOESM3_ESM.docx]

Meta-analysis of bone mineral density in adults with phenylketonuria

Júlio C. Rocha, Álvaro Hermida, Cheryl J. Jones, Yunchou Wu, Gillian E. Clague, Sarah Rose, Kaleigh B. Whitehall, Kirsten K. Ahring, André L.S. Pessoa, Cary O. Harding, Fran Rohr, Anita Inwood, Nicola Longo, Ania C. Muntau, Serap Sivri, François Maillot

# Supplementary information

Additional file 3: Fig. S2 Funnel plot for prevalence of BMD Z-score thresholds of: A) < -1.0, B) < -1.0 and ≥ -2.5, C) ≤ -2.0, and D) < -2.5 in adults with PKU on a Phe-restricted diet


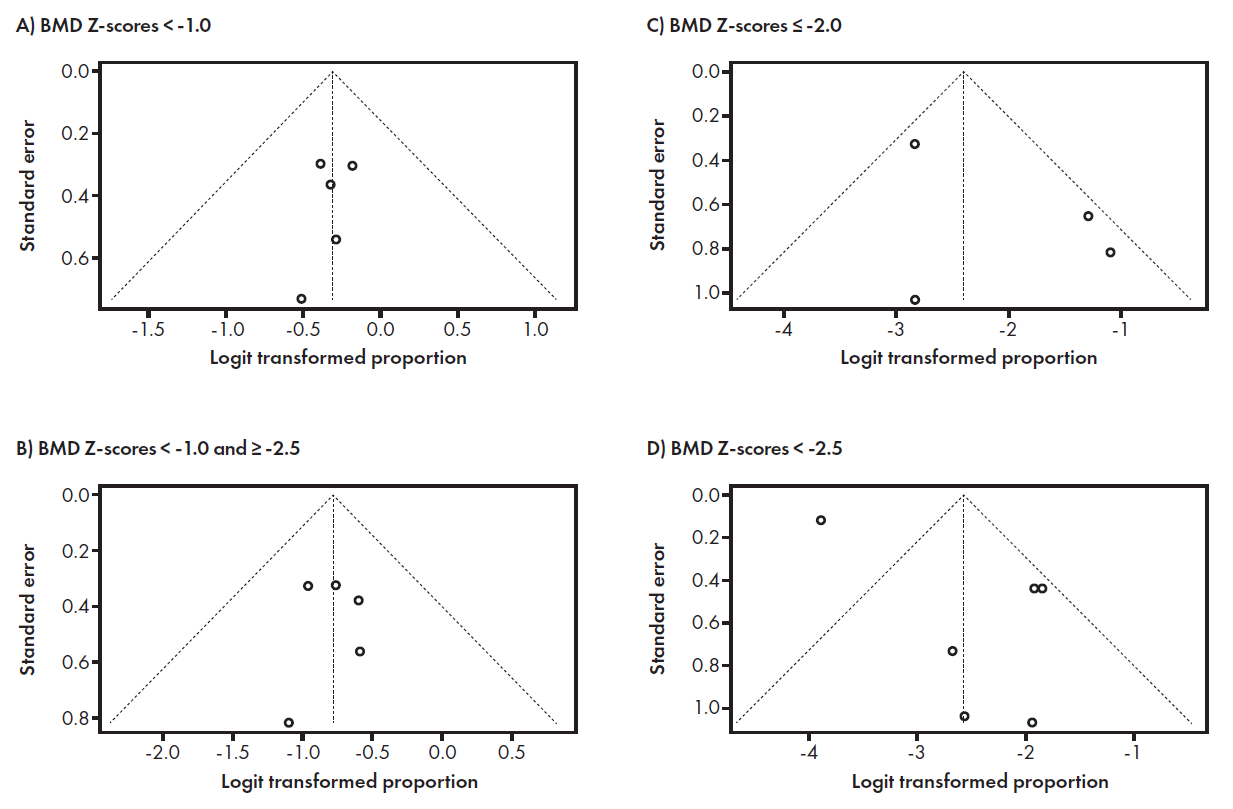


BMD, bone mineral density; CI, confidence interval; Phe, phenylalanine; PKU, phenylketonuria.
