# Supplementary material for: Meta-analysis of bone mineral density in adults with phenylketonuria
Source: Orphanet J Rare Dis. 2024 Sep 12;19:338. doi: 10.1186/s13023-024-03223-9 (PMC11391789; doi:10.1186/s13023-024-03223-9)
Supplement: Supplementary file 4 — Additional file 4. [file 13023_2024_3223_MOESM4_ESM.docx]

Meta-analysis of bone mineral density in adults with phenylketonuria

Júlio C. Rocha, Álvaro Hermida, Cheryl J. Jones, Yunchou Wu, Gillian E. Clague, Sarah Rose, Kaleigh B. Whitehall, Kirsten K. Ahring, André L.S. Pessoa, Cary O. Harding, Fran Rohr, Anita Inwood, Nicola Longo, Ania C. Muntau, Serap Sivri, François Maillot

# Supplementary information

Additional file 4: Fig. S3 Forest plot of lumbar spine BMD Z-scores for adults with PKU on a Phe-restricted diet versus a reference (non-PKU) population (BMD Z-score = 0) by A) decade of study publication (proxy for improvement in diet and supplements), B) controlled versus uncontrolled blood Phe levels, and C) gender


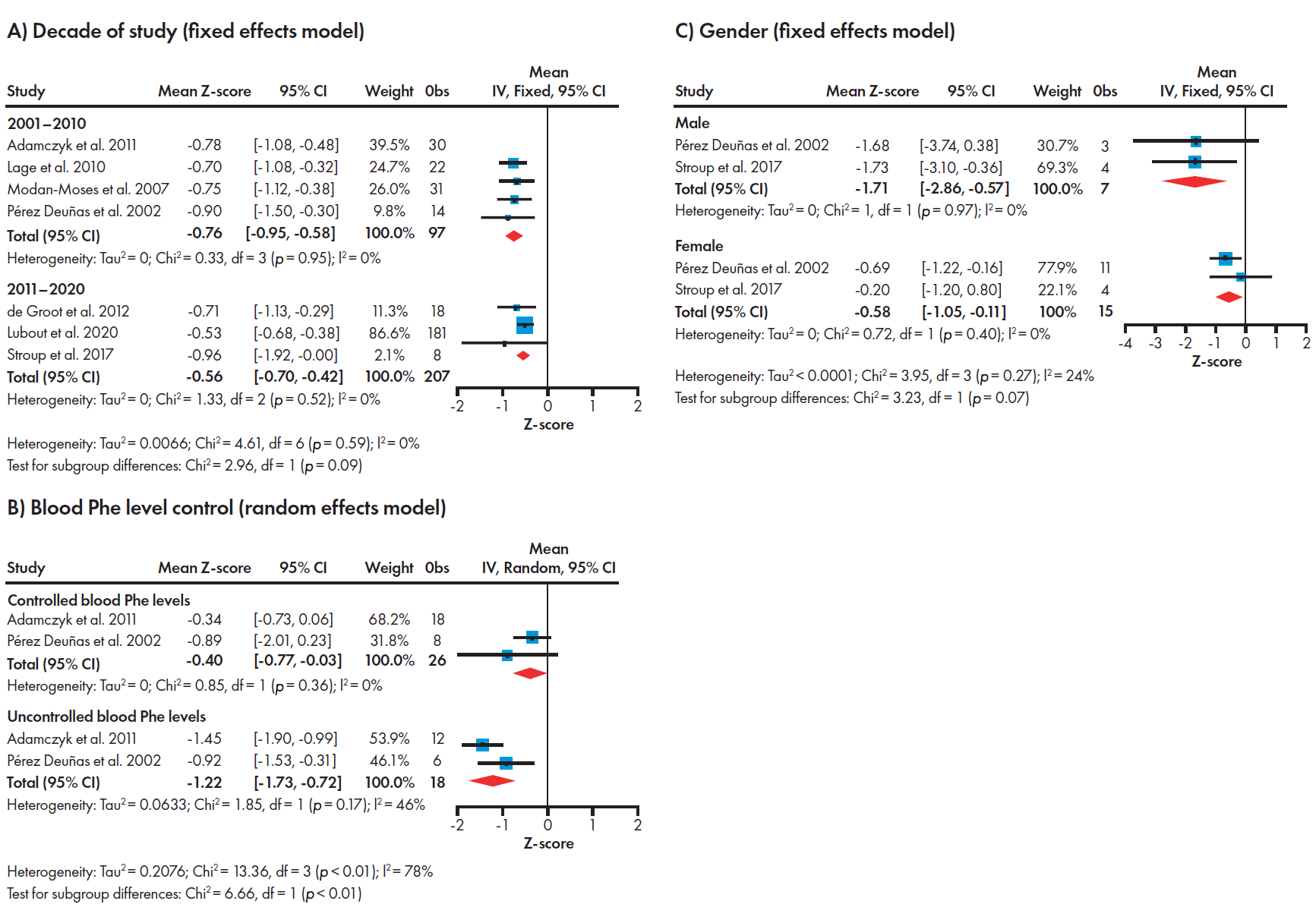


BMD, bone mineral density; CI, confidence interval; df, degrees of freedom; I^2^, heterogeneity; IV, inverse variance; Obs, observations; Phe, phenylalanine; PKU, phenylketonuria.

Effect size was estimated using either a fixed or random effects model based on the overall level heterogeneity score (considering both subgroups) [1].
