# Supplementary material for: Meta-analysis of bone mineral density in adults with phenylketonuria
Source: Orphanet J Rare Dis. 2024 Sep 12;19:338. doi: 10.1186/s13023-024-03223-9 (PMC11391789; doi:10.1186/s13023-024-03223-9)
Supplement: Supplementary file 5 — Additional file 5. [file 13023_2024_3223_MOESM5_ESM.docx]

Meta-analysis of bone mineral density in adults with phenylketonuria

Júlio C. Rocha, Álvaro Hermida, Cheryl J. Jones, Yunchou Wu, Gillian E. Clague, Sarah Rose, Kaleigh B. Whitehall, Kirsten K. Ahring, André L.S. Pessoa, Cary O. Harding, Fran Rohr, Anita Inwood, Nicola Longo, Ania C. Muntau, Serap Sivri, François Maillot

# Supplementary information

Additional file 5: Table S2 Femoral neck and total body BMD Z-scores for adults with PKU on a Phe-restricted diet versus the respective reference (non-PKU) population (BMD Z-score = 0) grouped by decade of study publication

| Bone location | Total no. of patients | Mean BMD Z-score (95% CI) | Heterogenerity (I^2^ test) | Subgroup difference, chi-square test |
| --- | --- | --- | --- | --- |
| **Femoral neck** | | | | |
| 2001–2010 [2, 3] | 53 | -1.10 (-1.86, -0.34)^a^ | 91%^b^ | *p* = 0.05 |
| 2011–2020 [4, 5] | 117 | -0.33 (-0.49, -0.16)^a^ |  |  |
| **Total body** | | | | |
| 2001–2010 [2, 6] | 61 | -0.51 (-0.70, -0.33) | 49%^c^ | *p* = 0.04 |
| 2011–2020 [4, 5] | 96 | -0.91 (-1.24, -0.58) |  |  |

^a^ Statistically significantly lower mean Z-score versus a reference (non-PKU) population. ^b^ Effect size was estimated using a random effects model based on the overall level heterogeneity score (considering both subgroups) [1]. ^c^ Effect size was estimated using a fixed effects model based on the overall level heterogeneity score (considering both subgroups) [1].

BMD, bone mineral density; CI, confidence interval; Phe, phenylalanine; PKU, phenylketonuria.

# References

1. Borenstein M, Hedges L, Higgins J, Rothstein H. *Introduction to meta-analysis*: John Wiley & Sons; 2011.

2. Modan-Moses D, Vered I, Schwartz G, et al. Peak bone mass in patients with phenylketonuria. *J Inherit Metab Dis*. 2007;30(2):202-8.

3. Lage S, Bueno M, Andrade F, et al. Fatty acid profile in patients with phenylketonuria and its relationship with bone mineral density. *J Inherit Metab Dis*. 2010;33 Suppl 3:S363-71.

4. Stroup BM, Sawin EA, Murali SG, Binkley N, Hansen KE, Ney DM. Amino acid medical foods provide a high dietary acid load and increase urinary excretion of renal net acid, calcium, and magnesium compared with glycomacropeptide medical foods in phenylketonuria. *J Nutr Metab*. 2017;2017:1909101.

5. Lubout CMA, Arrieta Blanco F, Bartosiewicz K, et al. Bone mineral density is within normal range in most adult phenylketonuria patients. *J Inherit Metab Dis*. 2020;43(2):251-58.

6. Adamczyk P, Morawiec-Knysak A, Płudowski P, Banaszak B, Karpe J, Pluskiewicz W. Bone metabolism and the muscle-bone relationship in children, adolescents and young adults with phenylketonuria. *J Bone Miner Metab*. 2011;29(2):236-44.
